# Transurethral Treatment of a Seminal Vesicle Cyst With Lithiasis: Case Report

**DOI:** 10.1155/cris/5599829

**Published:** 2025-05-11

**Authors:** Mohamadhusni Zarli, Joao G. Porto, Ruben Blachman-Braun, Hemendra N. Shah

**Affiliations:** ^1^Kiran C. Patel College of Osteopathic Medicine, Nova Southeastern University, Fort Lauderdale, Florida, USA; ^2^Desai Sethi Urology Institute, University of Miami, Miller School of Medicine, Coral Gables, Florida, USA

**Keywords:** case report, semen analysis, seminal vesicle cyst, seminal vesicle stones, transurethral seminal vesiculoscopy

## Abstract

Seminal vesicle cyst (SVC) is a rare condition that can arise from congenital or acquired causes. In this report, we describe the case of a 33-year-old male who was incidentally discovered to have SVC and seminal vesicle stones following complaints of abdominal pain. He presented to the emergency department with these symptoms, which prompted further investigation and diagnosis. Computerized tomography (CT) scan revealed diverticulitis and a fluid attenuation lesion within the right aspect of the prostate gland. He was referred to urology, and further evaluation showed a right SVC of 1.8 × 1.5 × 1.5 cm on magnetic resonance imaging (MRI). The patient underwent endoscopic drainage of the SVC via transurethral seminal vesiculoscopy (TRU-SVS) and holmium laser incision. Three stones ranging from 2 to 4 mm were identified and removed with N-gage basket from the seminal vesicle resulting in successful removal of the stones and restoration of the ejaculatory duct's patency. Follow-up visits showed resolution of pain and an improvement in semen volume. This case report highlights the importance of considering SVC as a differential diagnosis in male patients presenting with such symptoms. TRU-SVS is a feasible and effective treatment option for SVC and associated seminal vesicle stones.

## 1. Introduction

Seminal vesicle cyst (SVC) is an extremely uncommon condition, affecting fewer than 0.005% of individuals [1, 2]. SVCs may arise from either congenital or acquired causes. Congenital cysts are often associated with unilateral renal agenesis (known as Zinner syndrome) and heterotopic vas deferens, which are typically caused by malformation in the mesonephric duct. On the other hand, acquired cysts may result from inflammation, prostate surgeries, or ejaculatory duct obstructions, such as urolithiasis within the male reproductive system [1]. While most of these cysts are likely to be asymptomatic, they are typically identified in patients between their second and fourth decades of life. Symptoms tend to arise during the years of sexual activity when drainage is hindered by a malformed or narrowed duct system [3]. If present, individuals with SVC primarily have clinical symptoms of pelvic pain, lower urinary tract symptoms (LUTS), and prostatitis-like symptoms. Additionally, reduced volume of ejaculate, hematospermia, epididymitis, and infertility may manifest as the cysts can cause compression and cause obstruction of nearby structures such as vas deferens or ejaculatory duct [3]. In this report, we present a case of a 33-year-old male who was incidentally found to have an SVC that was causing pelvic pain. Upon workup, he was found to have low ejaculatory volume, and during endoscopic management of the cyst, small seminal vesicle stones were encountered.

## 2. Case Report

**Figure 1:**
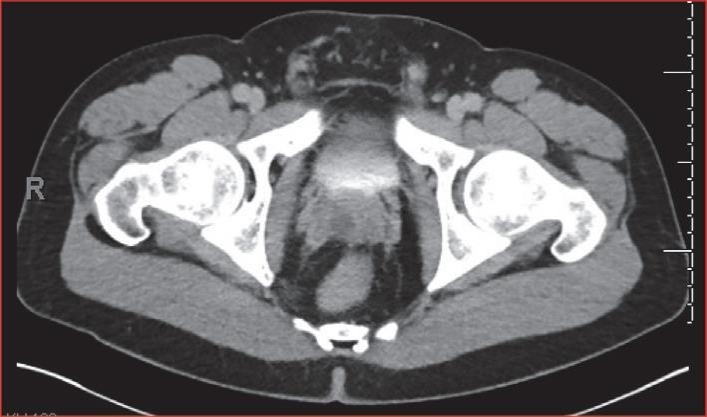
CT of pelvis reveals 1.7 cm fluid attenuation lesion within the right aspect of the prostate gland.

A 33-year-old male presented to the emergency department with complaints of abdominal pain. A full physical examination, urine testing, and computerized tomography (CT) scan of the abdomen were performed, revealing diverticulitis. Incidentally, it was found to have a 1.7 cm fluid attenuation lesion within the right aspect of the prostate gland and was referred to urology once diverticulitis resolved (Figure 1).

**Figure 2:**
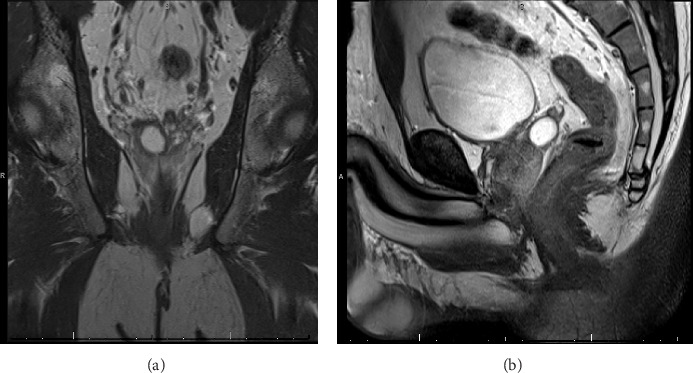
MRI of the prostate reveals right seminal vesicle cyst and low signal intensity. (A) Coronal view of the T2-weighted scan. (B) Sagittal view of the T2-weighted scan.

During the evaluation by the urologist, the patient reported occasional spasmodic pain in the lower abdomen, but denied any dysuria, hematuria, hematospermia, or any other LUTS. He was married and had no children, as he has not attempted or expressed a desire to conceive. He reported being sexually active with normal erections and no ejaculatory concerns. On examination, the prostate was soft, nontender, and without nodules. PSA was 0.35 ng/dL, semen analysis showed low volume of 1.0 mL, 7.2 pH, concentration of 25 10 × 6/mL, total motility of 40%, progressive motility of 40%, and total motile sperm count (TMSC) of 10. Transrectal ultrasound (TRUS) of the prostate was performed in the office showing an SVC and magnetic resonance imaging (MRI) was further performed confirming an SVC (Figure 2).

**Figure 3:**
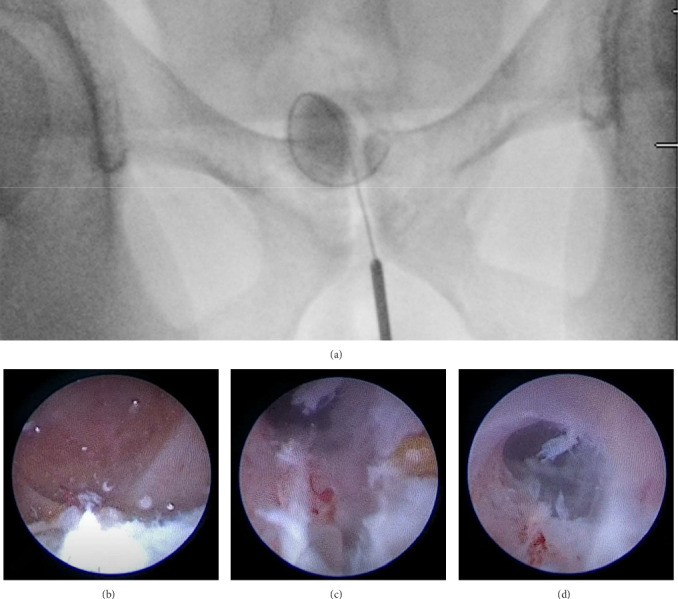
Intraoperative imaging of transurethral seminal vesiculoscopy with lithotripsy and incision of the right ejaculatory duct. (A) Fluoroscopy image after contrast was injected through semi-rigid ureteroscope into the right SV and a wire was placed into the SV. (B) Ejaculatory duct incision where the laser is increasing at the 5 o'clock position of the ejaculatory duct, a 365-micron laser fiber at 8 J and 8 Hz was used. (C) A stone fragment that was previously lasered has been irrigated. (D) Visualization of the ejaculatory duct after incision was performed.

Option of watchful waiting versus endoscopic management was discussed with the patient. Since he had intermittent spasmodic pelvic pain, he opted to undergo endoscopic drainage of the cyst after discussing management options. The patient underwent a cystoscopy with bilateral seminal vesiculography/vasography. During the procedure, the urethra and bladder were found to be normal, and two openings of the ejaculatory duct were identified. A TRUS was used to identify both seminal vesicles intraoperatively. They were cannulated with a 5-Fr Pollack catheter, and half-dilute contrast was injected under c-arm guidance. During seminal vesiculography, a narrow right ejaculatory duct with a 2 cm cystic dilatation of the right seminal vesicle was noted, which displaced the remaining right seminal vesicle medially. The left ejaculatory duct and seminal vesicle were normal. Seminal vesicoscopy was performed with an 8/9.5 Fr semi-rigid ureteroscope (Karl Storz, Tuttlingen, Germany) and narrow ejaculatory duct was incised at the 5 o'clock position using 365-micron laser fiber at setting of 8 J and 0.8 Hz. The dilated cyst was entered, and three stones of 2–4 mm were found in the SVC. These were moved with a zero-tip basket and then irrigated out of the bladder. Finally, the right seminal vesiculography confirmed a widely patent ejaculatory duct (Figure 3). The patient was discharged on the same day without complications.

During the 8-week postoperative follow-up visit, the patient reported that he no longer experienced pelvic pain after the surgery. Macroscopic hematospermia was observed during the postoperative course, which resolved completely within 2–3 weeks. He further reported that he did not notice a significant change in his postoperative ejaculatory characteristics. His repeat semen analysis at 8 weeks showed an improvement of semen volume to 1.3 mL post-surgery, 31 10 × 6/mL sperm concentration, total motility 42%, progressive motility 42%, and TMCS 17. The remaining parameters of the semen analysis remained within normal ranges. A pelvic MRI performed in the follow-up showed no remarkable differences. He remained asymptomatic at 6-month follow-up.

## 3. Discussion

The development of cysts within the seminal vesicles is uncommon and fewer than 100 cases have been reported. While a congenital SVC can be an isolated finding, developmental abnormalities of the seminal vesicles are often associated with upper urinary tract anomalies. About 66% of cases of congenital SVCs are found in conjunction with ipsilateral renal agenesis or dysgenesis. Other anomalies, such as ectopic ureteral insertion and agenesis of the vas deferens, have also been reported. In instances of bilateral SVC, up to 60% of patients may have autosomal dominant polycystic kidney disease [4]. In contrast, acquired cases of SVC are commonly caused by obstruction of the ejaculatory duct, often due to ascending urinary tract infections from the prostatic urethra into the seminal vesicles. Older populations are typically more affected by SVC and often associated with prostatitis, epididymitis, benign prostatic hyperplasia (BPH), and previous prostate surgery [1, 4]. In the case described here, the lack of additional anomalies and the identification of stones in the ejaculatory duct during surgery suggest that the cyst was acquired due to the narrow ejaculatory duct causing the stasis of seminal fluid and secondary stones.

Although SVCs are usually benign, malignant tumors of the seminal vesicles, such as adenocarcinomas and sarcomas, should also be considered as a differential diagnosis [5].

Like SVCs, seminal vesicle calculi are a rare occurrence, with only 213 reported cases found in a systematic review spanning from 1928 to 2016 [6]. Lithiasis in the seminal vesicle was initially reported by White, and their pathogenesis is thought to involve a combination of factors, including obstructed ejaculatory ducts, anatomical abnormalities, infective processes, and urinary reflux. The presence of seminal vesicle calculi is often associated with chronicity in male accessory gland infections, while calcification has been linked to conditions such as diabetes and chronic inflammation caused by diseases like tuberculosis and prostatitis [6, 7].

Patients with seminal vesicle calculi, similar to those with isolated SVC, may not experience any symptoms or may present with peritoneal or testicular pain, painful ejaculation, low ejaculate volumes, hematospermia, or infertility [6]. TRUS, CT, and MRI are used to identify abnormalities in the seminal vesicles and for the diagnosis of both SVC and seminal vesicle stones, with TRUS being the gold standard imaging modality. TRUS has a high sensitivity for detecting prostate and seminal vesicle abnormalities and is a cost-effective investigation that is easily available in clinical settings [6, 7]. Although TRUS is cheaper and more readily available than other imaging modalities, seminal vesiculography may be necessary to confirm the presence of seminal vesicle obstruction and stones [7]. In this case, TRUS was able to identify the SVC, but the diagnosis of the three seminal vesicle calculi was not made until seminal ventriculoscopy was performed.

Over the years, SVCs have been considered to be benign and typically managed conservatively with close follow-up and nonsurgical treatments. However, clinicians have long recognized the association between SVCs can result in seminal outlet obstruction and can grow to a significant size that can progress to cause infertility, pelvic pain, hydronephrosis, and even rectal obstruction. This has led to the consideration of surgical interventions as effective therapies for symptomatic SVCs since the late 19th century. Both transperitoneal laparoscopic unroofing (TLU) and fenestration under seminal vesiculoscopy (FUSV) are effective surgical therapies for removing SVC lesions. However, the limited data available does not support the notion that either procedure has a positive effect on sperm quality or fertility. One study included nine patients who underwent preoperative and postoperative semen analysis, found no significant improvement in total sperm count (TSC) or TMSC compared to preoperative levels [8]. In this case presented, the patient showed a slight increase in the ejaculatory volume from 1 mL pre-surgery to 1.3 mL post-surgery. Despite this improvement, the volume remained below 1.5 mL, highlighting that while there was an improvement in semen volume, it still falls short of the normal reference range, underscoring the partial nature of the improvement. Additionally, a follow-up cystoscopy was not performed as the patient reported no significant symptoms during follow-up visits, aligning with the current practice of avoiding invasive follow-up procedures in asymptomatic patients.

The recommended treatment for symptomatic seminal vesicle calculi is surgical intervention. TRU-SVS has shown to be a safe, minimally invasive surgical procedure with lower complications than transurethral resection of ejaculatory duct and significant improvement in sperm counts and ejaculation volume [6, 9]. Endoscopic removal is a minimally invasive procedure that can be performed in symptomatic seminal vesicle calculi; transurethral approach has shown to be effective in treating seminal vesicle calculi. Endoscopic removal using TRU-SVS has shown to be safe, with fewer complications, significant symptom relief, increased ejaculatory volume, and potential improvement in semen parameters, thereby enhancing fertility outcomes compared to traditional methods. The choice of surgical protocol depends on the location and size of the stone [6]. For the presented patient, three stones were irrigated out during TRU-SVS.

Building on these advancements in minimally invasive techniques, the application of laser therapy in related urological conditions, such as ejaculatory duct obstruction and prostatic utricle cysts, is an evolving area of interest. While laser use is not yet described specifically for seminal vesicle calculi, studies have documented the efficacy, safety, and outcomes of laser-assisted approaches in managing these conditions [10–16]. The use of either holmium or thulium laser is described in literature to excise or vaporize the obstructing tissue in a precise and controlled manner with reduced bleeding during surgery [13, 16]. Laser treatment has been shown to cause improved semen parameters, such as sperm motility and count, leading to better fertility outcomes [12].

## 4. Conclusion

SVCs and seminal vesicle calculi are rare causes of pelvic pain in males, which may be overlooked due to their infrequency and lack of physical examination findings. TRUS and MRI are effective imaging modalities for diagnosis, while surgical intervention is recommended for treatment. TRU-SVS is a safe and minimally invasive procedure with significant improvements in semen volume and pelvic pain.

## Data Availability

The clinical data used to support the findings of this case report are included in this case report/series.
